# Gout, tophi and the wonders of NETs

**DOI:** 10.1186/s13075-014-0431-2

**Published:** 2014-08-30

**Authors:** David S Pisetsky

**Affiliations:** Medical Research Service, Durham VA Medical Center, Duke University Medical Center, Durham, NC 27710 USA

## Editorial

‘If I didn’t work on lupus, I would work on gout,’ I tell my fellow as we stand outside the room of a patient with an acute flare, his knee red and swollen. I have made this assertion many times, eliciting surprise if not mystification since lupus, the main focus of my research, and gout seem polar opposites. Lupus is a chronic antibody-mediated disease while gout is an acute cytokine-mediated disease.

What I say next would prompt genuine concern about my mental well-being. ‘And, if I worked on gout, I would study the tophus,’ I say decisively.

Lest you think I am going off the rails, I believe that study of the tophus holds important insights for immunology. While interest in the tophus has surged recently as an outcome measure in treatment, it is often an afterthought, infrequently analyzed. Few studies have characterized tophi by modern immunofluorescence or electron microscopic techniques. Nevertheless, recent studies indicate that the tophus may be a goldmine to find real gems on immune regulation. Such studies are also strengthening what to me has always been quite simple and obvious: the lupus–gout connection.

Despite differences in clinical course, both lupus and gout concern the response to single molecules. These molecules are linked biochemically and, indeed, one is the product of the other. For lupus, the molecule is DNA; for gout, it is uric acid. DNA is built of purines (and pyrimidines), with uric acid a byproduct of purine degradation. Furthermore, both DNA and uric acid are damage-associated molecular patterns, released during cell death to signal danger and trigger inflammation. Hence, my attraction to these diseases.

It is in the context of immune regulation that the tophus takes center stage. Monosodium urate (MSU), the crystalline salt form of uric acid, is one of the most proinflammatory chemicals ever found. In the tophus core, MSU should be present at enormous concentrations, with milk of urate essentially a suspension of gout crystals. Yet, despite the clear and present danger of so much MSU – a veritable immunological time bomb – the tophus is a remarkably placid structure. Even when a tophus breaks down and milk of urate cascades out, the result appears benign, the surrounding skin neither red nor raw.

Even a novice investigator has to ask what is going on? By analogy to Newton’s third law of motion, what is the equal and positive reaction that pushes back on the gout crystals’ action? As shown in a provocative study by Schauer and colleagues in the journal *Nature Medicine*, the neutrophil itself may perform the push back, pacify the tophus and shut down a gout attack just as it fires one up. This downregulatory mechanism involves an unexpected player: the neutrophil extracellular trap (NET).

A NET is a mesh or web-like structure formed by the mixture of chromatin with the contents of neutrophil granules including antibacterial proteins such as myeloperoxidase, neutrophil elastase and cathelicidins. In a process involving the generation of reactive oxygen species by NAPH oxidase (Nox), the NET structure is extruded into the extracellular space to kill bacteria or fungi ensnared in this structure. Importantly, NETs are present in the tophus.

Given its essential role in host defense, why should a NET control inflammation? The intriguing studies by Schauer and colleagues provide a neat explanation. Thus, the proteases that stud the NET can also degrade cytokines and hence curtail their action. With neutrophils present in high concentrations, as could occur in gouty inflammation, the NETs can aggregate to increase the concentration of serine proteases to exert a counter-regulatory action. Such aggregated NETs can degrade a wide array of cytokines such as IL-6, MIP-1α, IL-1β and TNF-α, all powerful players in gout pathogenesis. Since NETs are present in the tophi, as shown by immunofluorescence microscopy, they could represent a counterforce that keeps the structure quiescent by degrading cytokines, perhaps produced by macrophages.

An important set of experiments in the study by Schauer and colleagues used murine models of gouty inflammation *in vivo*. These studies involved *ncf1*^*++*^ mice, which are a model of chronic granulomatous disease. These mice are deficient in NETosis because cells cannot produce reactive oxygen species by Nox2 because of a mutation in neutrophil cytosolic factor 1 (encoded by *Ncf*1). In these studies, administration of MSU crystals into air pouches of *Ncf1*^*++*^ mice led to higher levels of cytokines than those of wild-type mice, while administration of MSU crystals into the foot pads of these NETosis-deficient animals had a more chronic course that lasted for weeks. Furthermore, in an air pouch model in wild-type mice, DNase treatment reduced the NET aggregation and modulated cytokine production. Together, these studies suggest that NETosis and the aggregation of NETs can restrain inflammation or promote resolution in gout by degrading proinflammatory cytokines.

The role of NETs in shaping the microenvironment of the tophus is intriguing but much work remains to be done to validate this hypothesis. More histopathology is definitely needed since the few recent studies on this subject indicate a striking paucity of neutrophils in the tophus; on the other hand, analysis of proteins in the tophus indicates the presence of myeloperoxidase and histones, both NET constituents. Perhaps neutrophils only transiently populate the tophus, lay down the NETs and then disappear. While large datasets – whether genomic, transcriptomic or proteomic – are the vogue, it is refreshing to see pathological studies – the demonstration of NETs near the tophus core – producing such exciting insights. These insights in turn lead to a reconceptualization of the NET – from Wild West desperado into the sheriff bringing law and order.

While my heart remains wedded to lupus, I cannot help but feel a strange pull emanating from the tophus. As the great Jacques Cousteau said: ‘The Sea, once it casts its spell, holds one in its net of wonder forever’. I feel the same way about the tophus and I am afraid that I too will be entranced by a wondrous place and become trapped in an entangling net.

## Suggested reading

Brinkmann V, Zychlinsky A: **Neutrophil extracellular traps: is immunity the second function of chromatin?***J Cell Biol* 2012, **198:**773**–**783.

Dalbeth N, Clark B, Gregory K, Gamble GD, Doyle A, McQueen FM: **Computed tomography measurement of tophus volume: comparison with physical measurement.***Arthritis Rheum* 2007, **57:**461**–**465.

Dalbeth N, Pool B, Gamble GD, Smith T, Callon KE, McQueen FM, Cornish J: **Cellular characterization of the gouty tophus: a quantitative analysis.***Arthritis Rheum* 2010, **62:**1549**–**1556.

Kaneko K, Iwamoto H, Yasuda M, Inazawa K, Yamaoka N, Fukuuchi T, Tamura Y, Uchida S, Mawatari K, Nakagomi K, Yamada Y, Fujimori S: **Proteomic analysis to examine the role of matrix proteins in a gouty tophus from a patient with recurrent gout.***Nucleosides Nucleotides Nucleic Acids* 2014, **33:**199**–**207.

Manger B, Lell M, Wacker J, Schett G, Rech J: **Detection of periarticular urate deposits with dual energy CT in patients with acute gouty arthritis.***Ann Rheum Dis* 2012, **71:**470**–**472.

Melzer R, Pauli C, Treumann T, Krauss B: **Gout tophus detection – a comparison of dual-energy CT (DECT) and histology.***Semin Arthritis Rheum* 2014, **43:**662**–**665.

Palmer DG, Hogg N, Denholm I, Allen CA, Highton J, Hessian PA: **Comparison of phenotype expression by mononuclear phagocytes within subcutaneous gouty tophi and rheumatoid nodules.***Rheumatol Int* 1987**, 7:**187**–**193.

Schauer C, Janko C, Munoz LE, Zhao Y, Kienhofer D, Frey B, Lell M, Manger B, Rech J, Naschberger E, Holmdahl R, Krenn V, Harrer T, Jeremic I, Bilyy R, Schett G, Hoffmann M, Herrmann M: **Aggregated neutrophil extracellular traps limit inflammation by degrading cytokines and chemokines.***Nat Med* 2014, **20:**511**–**517.

Schweyer S, Hemmerlein B, Radzun HJ, Fayyazi A: **Continuous recruitment, co-expression of tumour necrosis factor-alpha and matrix metalloproteinases, and apoptosis of macrophages in gout tophi.***Virchows Arch* 2000**, 437:**534**–**539.

Steiger S, Harper JL: **Neutrophil cannibalism triggers transforming growth factor β1 production and self regulation of neutrophil inflammatory function in monosodium urate monohydrate crystal-induced inflammation in mice.***Arthritis Rheum* 2013, **65:**815**–**823.

